# PGAweb: A Web Server for Bacterial Pan-Genome Analysis

**DOI:** 10.3389/fmicb.2018.01910

**Published:** 2018-08-21

**Authors:** Xinyu Chen, Yadong Zhang, Zhewen Zhang, Yongbing Zhao, Chen Sun, Ming Yang, Jinyue Wang, Qian Liu, Baohua Zhang, Meili Chen, Jun Yu, Jiayan Wu, Zhong Jin, Jingfa Xiao

**Affiliations:** ^1^Computer Network Information Center, Chinese Academy of Sciences, Beijing, China; ^2^BIG Data Center, Beijing Institute of Genomics, Chinese Academy of Sciences, Beijing, China; ^3^CAS Key Laboratory of Genome Sciences and Information, Beijing Institute of Genomics, Chinese Academy of Sciences, Beijing, China; ^4^College of Life Sciences, University of Chinese Academy of Sciences, Beijing, China; ^5^Lymphocyte Nuclear Biology, National Institute of Arthritis and Musculoskeletal and Skin Diseases, National Institutes of Health, Bethesda, MD, United States; ^6^Department of Computer Science and Engineering, The Pennsylvania State University, University Park, PA, United States; ^7^Office of General Affairs, Chinese Academy of Sciences, Beijing, China; ^8^Center of Scientific Computing Applications and Research, Chinese Academy of Sciences, Beijing, China

**Keywords:** web server, bacterial pan-genome analysis, genome dynamics visualization, genome structure interpretation, orthologous gene identification

## Abstract

An astronomical increase in microbial genome data in recent years has led to strong demand for bioinformatic tools for pan-genome analysis within and across species. Here, we present PGAweb, a user-friendly, web-based tool for bacterial pan-genome analysis, which is composed of two main pan-genome analysis modules, PGAP and PGAP-X. PGAweb provides key interactive and customizable functions that include orthologous clustering, pan-genome profiling, sequence variation and evolution analysis, and functional classification. PGAweb presents features of genomic structural dynamics and sequence diversity with different visualization methods that are helpful for intuitively understanding the dynamics and evolution of bacterial genomes. PGAweb has an intuitive interface with one-click setting of parameters and is freely available at http://PGAweb.vlcc.cn/.

## Introduction

The advancement of sequencing technology and assembly tools has facilitated fast acquisition of genome sequences of various species over the past two decades. Tettelin et al. (2005) coined the concept of the pan-genome in. The pan-genome encompasses the entire repertoire of genes accessible to a studied phylogenetic clade or a given species, which is divided into the core genome (genes shared by all strains), dispensable genes (genes shared only by a subset of the strains), and strain-specific (unique) genes. Pan-genomics has far-reaching significance (Vernikos et al., 2015; Xiao et al., 2015), especially in bacterial research, and has been applied to many fields, such as species evolution (Lefebure and Stanhope, 2007; Rasko et al., 2008), investigation of pathogenic and drug resistance mechanisms (D’Auria et al., 2010; Hu et al., 2011; Zhang et al., 2011), vaccine development (Maione et al., 2005; Serruto et al., 2009), host and environment adaptability (Saenz et al., 2007; Sugawara et al., 2013; Tian et al., 2016), and population structures (Donati et al., 2010).

To make bacterial pan-genome analysis convenient and efficient, several tools, online databases, and web servers have been developed over the past two decades (Vernikos et al., 2015; Xiao et al., 2015). PGAP (Zhao et al., 2012), GET_HOMOLOGUES (Contreras-Moreira and Vinuesa, 2013), ITEP (Benedict et al., 2014), PanGP (Zhao et al., 2014), Roary (Page et al., 2015), and panX (Ding et al., 2018) were introduced as pan-genome analysis tools for clustering orthologous genes, identifying single nucleotide polymorphisms (SNPs), constructing phylogenies, function-based searching or analysis, and gene curation. However, they all are command-line tools demanding basic programming skills and with lack of in-depth inference and visualization of results. PGAT (Brittnacher et al., 2011), as a web-based database, provides many useful functions, such as predicting protein families, identifying SNPs, and providing analysis tools for Kyoto Encyclopedia of Genes and Genomes (KEGG) pathway and other genome annotations, but a limited number of species are integrated and users’ own data cannot be uploaded and analyzed. PanWeb (Pantoja et al., 2017), PANNOTATOR (Santos et al., 2013), and PanCGHweb (Bayjanov et al., 2010) are web servers for pan-genome analysis, but they all suffer from some flaws, such as limited functions or working only with microarray data.

In order to perform bacterial pan-genome analysis more efficiently, we have developed a user-friendly online tool, PGAweb, based on our previously published software, PGAP (Zhao et al., 2012), PanGP (Zhao et al., 2014), and PGAP-X (Zhao et al., 2018). PGAP and PanGP have been reported as the well-used toolkits for pan-genome analysis (Vernikos et al., 2015; Xiao et al., 2015; Zekic et al., 2018). To enhance interpretation and visualization of genome dynamics, we have integrated our newly developed software PGAP-X into PGAweb (Zhao et al., 2018). Based on these three tools, PGAweb serves as a fast pan-genome analysis web server with some great improvements. In brief, PGAweb has a user-friendly, web-based interface, one-click input data submission, and smooth and efficient data analysis, not only providing diverse methods for identifying orthologous genes, but also presenting genomic diversity with different visualization methods and genomic dynamics information.

## Methods and Implementation

**FIGURE 1:**
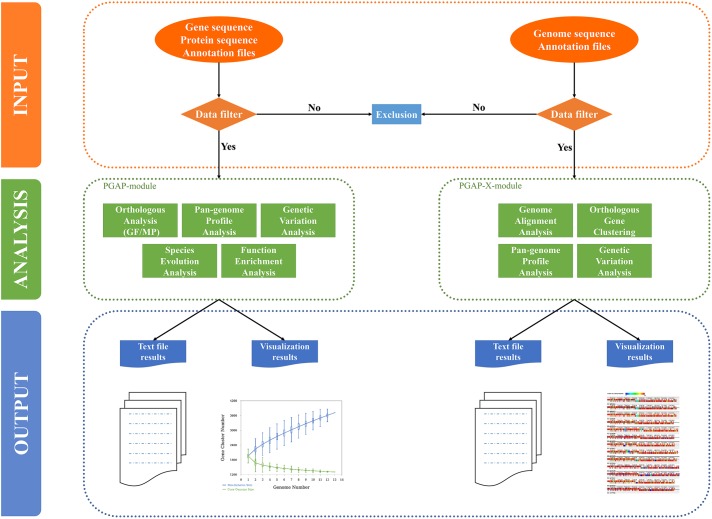
Organization of PGAweb. All analysis steps are grouped into three parts: data input, data analysis, and output-visualization (shown with different color codes and separated by dashed lines). The input data of each module will be checked. The filtered data will be used in the subsequent analysis. Green boxes report the analysis functions of each module. For the output, PGAweb provides one text file corresponding to a graph (genetic variation analysis in the PGAP module only provides tabular text).

PGAweb consists of two core modules, PGAP and PGAP-X. PGAP supports five main analytic functions including orthologous clustering, pan-genome profiling, sequence variation analysis, species phylogeny, and gene functional classification. PGAP-X performs four comparative genomics assignments: genome-wide sequence alignment, orthologous clustering, pan-genome profiling, and sequence variation analysis (**Figure 1**). Compared with PGAP, PGAP-X introduces a novel algorithm for orthologous cluster identification, which can distinguish paralogs based on conserved genomic location from a genome alignment. PGAP-X also supports a visualization function for genome-wide sequence alignment, genome structure variation, and orthologous mapping based on sequence conservation. Users are able to choose one of the modules according to their research objectives.

### PGAP Module

In this module, two different methods, MultiParanoid (MP) and GeneFamily (GF), are provided for orthologous clustering. In the MP method, first, orthologs between each pair of strains are identified by InParanoid (Remm et al., 2001; Ostlund et al., 2010); MP is then invoked to search for gene clusters among multiple strains based on the result of pairwise orthologous clusters from Inparanoid (Alexeyenko et al., 2006). With the GF method, all the protein sequences from the input data are first mixed together and an all vs. all sequence alignment is performed by the BLASTALL program; then, the MCL algorithm (Enright et al., 2002; Van Dongen, 2008) is directly invoked to cluster the filtered BLAST results. To detect the relationships between the numbers of genomes and pan-genome size or core gene clusters, pan-genome profiles are calculated based on orthologous clustering from all strains with the Heaps’ law or exponential model (Tettelin et al., 2005; Rasko et al., 2008). Genetic variation analysis and gene functional classification are also performed in this module. For each gene cluster, mutations, and indels are detected by protein sequence alignment of each cluster using MAFFT (Katoh and Toh, 2010) and then the aligned amino acid sequences are converted into corresponding codon-based nucleotide sequences. Functional classification analysis of gene clusters includes description of each cluster’s function and Clusters of Orthologous Groups (COG) classification (Tatusov et al., 2000) according to uploaded annotation files. The function description with highest frequency excluding “hypothetical protein” for all genes in each cluster is taken as the description of the cluster. For further elucidating genomic diversity, species evolution analysis is performed by two algorithms [Neighbor-joining and Unweighted Pair Group Method with Arithmetic Mean (UPGMA)] using PHYLIP (Felsenstein, 1989) based on two types of data: gene gain/loss matrix or SNPs in core gene clusters. The maximum-likelihood algorithm is only used in the analysis based on SNPs in core gene clusters.

### PGAP-X Module

In this module, a new in-house algorithm is used for orthologous gene clustering (Zhao et al., 2018). Whole-genome sequences or pseudo-chromosome sequences are aligned by progressiveMauve (Darling et al., 2010). In visualizing alignment results, colored blocks are used to display homologous DNA fragments across strains, which highlight conserved regions, strain-specific blocks, and recombination events including insertions, inversions, and deletions on a large scale. The distribution of orthologous genes in each conserved block is displayed in different colors that represent degrees of conservation for each gene. A pan-genome profile is calculated based on the same methods as in the PGAP module. Genetic variation analysis among pairwise or multiple genomes is presented. In this part, two vital parameters, variation frequency (*f*), and variation number (*n*) in 1 kb regions, are used to filter out high-substitution regions. Regions with the following traits will be identified as high-substitution regions: (a) no fewer than *n* substitution sites in the region; (b) variation frequency in the region is more than *f*; (c) the interval between any two substitution sites is less than 1/*f*. The high-substitution regions are marked on the genome structure. For pairwise variation analysis, a reference genome and a query genome should be selected.

### Implementation

The web server is compatible with many platforms, including Safari, Firefox, Opera, Chrome, and Internet Explorer, by using Bootstrap Framework^1^ as the front-end program. It adopts HTML5 and CSS3 protocols and uses D3.js^2^ and Echarts^3^ for result interaction. The server back-end adopts an Express Framework^4^ for Node.js MongoDB^5^ is used for the storage of related information. Docker^6^ is used as a container for packaging codes and software, which improves compatibility, portability, and safety. This website is open to all users, and login is not required.

## Workflow of PGAweb

### Step 1: Module Selection

**FIGURE 2:**
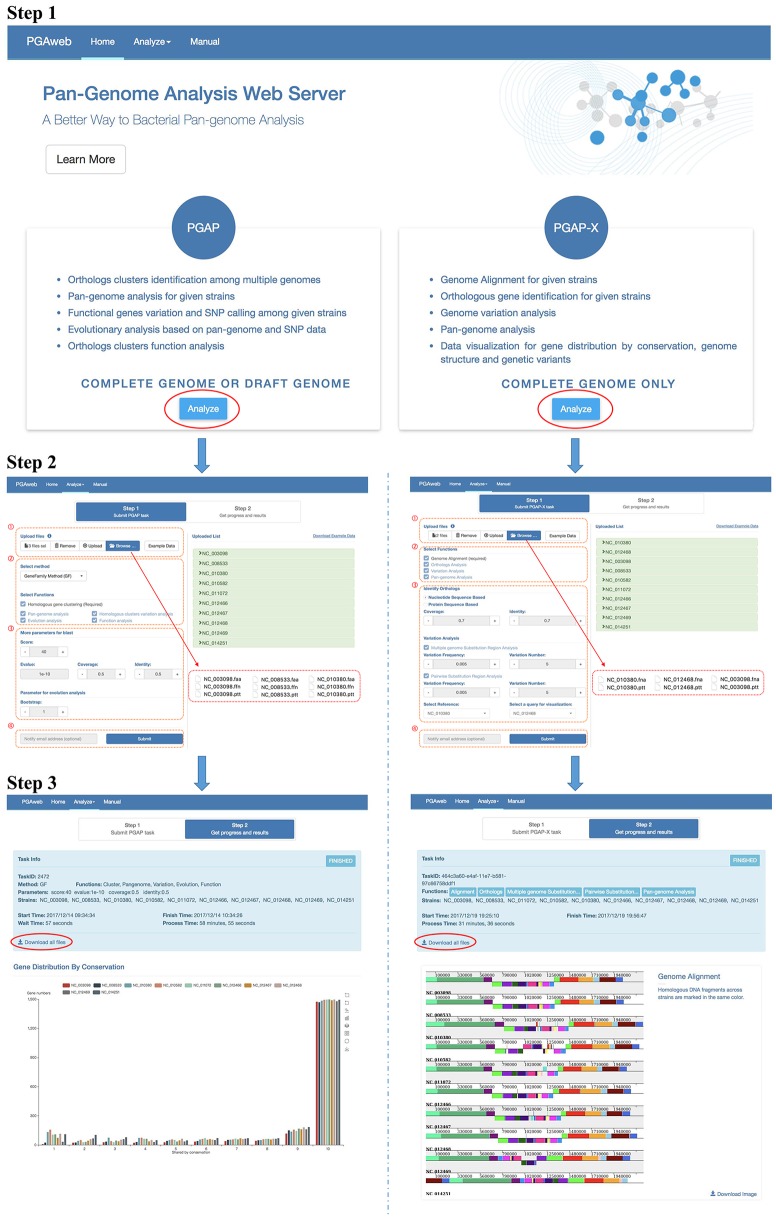
Step by step workflow of PGAweb. Step 1: Module selection. Step 2: Input data upload and parameter setting. (1) Data browse and upload, (2) method and function selection, (3) parameter setting, and (4) fill in e-mail address and submit job. Step 3: Results download.

The general workflow of PGAweb is shown in **Figure 2**. The first step is module selection for pan-genome analysis according to data type and research objectives. Two different pan-genome analysis modules are integrated in PGAweb. The PGAP module supports both draft and complete genomes and provides five main results, including orthologous clustering, pan-genome profiling, sequence variation analysis, species phylogeny, and gene functional classification. Most results generated by the PGAP module are text files. Detailed information can be found in these text files for in-depth pan-genome analysis. The PGAP-X module only supports analysis of complete genomes. The PGAP-X module’s capabilities include genome alignment, orthologous gene identification, genome variation detection, and pan-genome profile analysis. PGAP-X provides an in-house algorithm that can distinguish paralogous genes from orthologous genes based on conserved genomic location. The visualization function for genome structure conservation and gene distribution is integrated in the PGAP-X module.

### Step 2: Input Data Upload and Parameter Setting

This step contains four procedures (**Figure 2**). First, specific input data for pan-genome analysis should be selected and uploaded. The PGAP module requires three types of files for each strain: nucleotide and protein sequences as well as annotations. Four input formats are selectable: (1) NCBI new data format: .cds_from_genomic.fna.gz, protein.faa.gz, and .feature_table.txt.gz extensions. These files can be downloaded from ftp://ftp.ncbi.nlm.nih.gov/genomes/all/. (2) NCBI old data format: .ffn, .faa, and .ptt extensions. These files can be downloaded at ftp://ftp.ncbi.nlm.nih.gov/genomes/archive/old_genbank/. (3) PGAP raw data format: .nuc, .pep, and .function extensions. These data may come from the user’s own laboratory..nuc files store nucleotide sequences in FASTA format; .pep files store corresponding protein sequences in FASTA format; .function files store annotations with three columns including gene name, COG classification, and function description. (4) NCBI GenBank (full) format: .gb extension. In addition, each protein sequence should have its corresponding nucleotide sequence and annotation information, while the length of the nucleotide sequence minus 3 (excluded stop codon) must be equal to three times the corresponding protein length. All uploaded files are auto-transformed to corresponding PGAP raw data format and criterion checking is performed. The PGAP-X module needs a complete genome sequence and annotation information for each strain. The input files should have .fna and .ptt extensions. More detailed description is available at http://pgaweb.vlcc.cn/doc. The second procedure is the selection of the orthologous gene identification method and pan-genome analysis functions. In the PGAP module, either the GF method or the MP method can be chosen for orthologous gene identification. The GF method is fast, but less accurate compared with the MP method. The GF method is suitable choice for handling more than 50 genomes with consideration of calculation time. In the PGAP-X module, there is only one method for orthologous gene identification. After selection of gene identification method, users need to select the desired functions for pan-genome analysis. Homologous gene clustering for the PGAP module and genome alignment for the PGAP-X module is required. Third, parameters for selected functions need to be set. Finally, users may provide e-mail addresses for obtaining notification of finished results and click the submit button for running PGAweb.

### Step 3: Output Description

The output of the PGAP module is composed of five parts: orthologous clustering, pan-genome profile, evolutionary analysis, sequence variation analysis, and functional classification. For orthologous clustering, the results include an intuitive figure showing statistical gene distribution by conservation and two downloadable tabular files (1.Gene_Distribution_By_Conservation.txt and 1.Orthologs_Cluster.txt). For the pan-genome profile, the relationships between the numbers of genomes and pan-genome size or core gene clusters are calculated. Whether a pan-genome is open or closed can be easily inferred from visual results. Pan-genome profiles provide indirect indications of lifestyle and opportunity for multiple mechanisms of DNA exchange with the global microbial gene pool of the specific species. Multiple algorithms for bacterial evolutionary analysis are provided to improve the accuracy of phylogenetic inference, especially for closely related species. All phylogenetic trees calculated by PHYLIP are output as .tree files, and the user can use visualization software to view the phylogenetic trees. Files with .outfile extension give users a simple view of the phylogenetic trees. For sequence variation analysis, information on positions with variation, amino acid types, nucleotide types, and variation types (indels, and synonymous and non-synonymous mutations) are presented in tabular file 3.CDS.variation.txt. All statistical results of functional classification of core genes, dispensable genes, and strain-specific genes are dynamically displayed and compared. This result is useful for speculation of strain-specific physiological function and metabolic mechanism. Moreover, the actual gene lists (5.Orthologs_Core_Cluster_COG_Distribution.list.txt, 5.Orthologs_Dispensable_Cluster_COG_Distribution.list.txt, and 5.Orthologs_specific_Cluster_COG_Distribution.list.txt) used in the functional classification can be downloaded for in-depth analyzes.

Four parts of the pan-genome analysis results are showed in the PGAP-X output. Complete genome or pseudo-chromosome sequences are aligned in PGAP-X. In visualizing the alignment result, colored blocks are used to display homologous DNA fragments across strains, which highlight conserved regions, strain-specific blocks, and recombination events including insertions, inversions, and deletions on a large scale. The orthologous gene distribution in each conserved block is displayed in different colors that represent degrees of conservation for each gene. Genomic diversity of the bacterial population and differences in gene content are well depicted, and even horizontal gene transfer (HGT) or homologous recombination events are easily identified. Meanwhile, multiple-genome substitution and pairwise substitution regions are analyzed in PGAP-X. The high-substitution regions are marked on the genome structure with blue bars. For pairwise substitution region analysis, a reference strain must be selected; all sites of variation among paired genomes can be detected and reported in output text files but only high-substitution regions of selected strains are displayed. The influence of selective pressure on each genomic region or gene can be intuitively showed. Some highly variable regions may be involved in pathogenicity islands (PAIs) or related to strain adaptability. This may be used for SNP-based barcode design and gene selection for multilocus sequence typing (MLST). For the pan-genome profile, the result is the same as shown in the PGAP module.

## Case Study

To evaluate the performance of PGAweb, we carried out a case study for *Streptococcus suis*. The two modules, PGAP and PGAP-X, were used with default parameters (GF method was selected to identify orthologous genes in PGAP). The PGAweb results are described as follows.

Thirteen previously studied *S. suis* genomes (Zhang et al., 2011) were reanalyzed by using PGAweb. The genome sequence data can be downloaded easily from NCBI ftp^7^ . *S. suis* is a gram-positive pathogen that usually causes zoonosis (Staats et al., 1997). Thirty-three serotypes are classified based on the composition of their capsular polysaccharides. These strains are classified into seven serotypes including serotype 1 (NC_017950), serotype 1/2 (NC_017619), serotype 2 (NC_009442, NC_009443, NC_017622, NC_012926, NC_017617, NC_012925, and NC_012924), serotype 3 (NC_015433), serotype 7 (NC_017620), serotype 9 (NC_017621), and serotype 14 (NC_017618).

**FIGURE 3:**
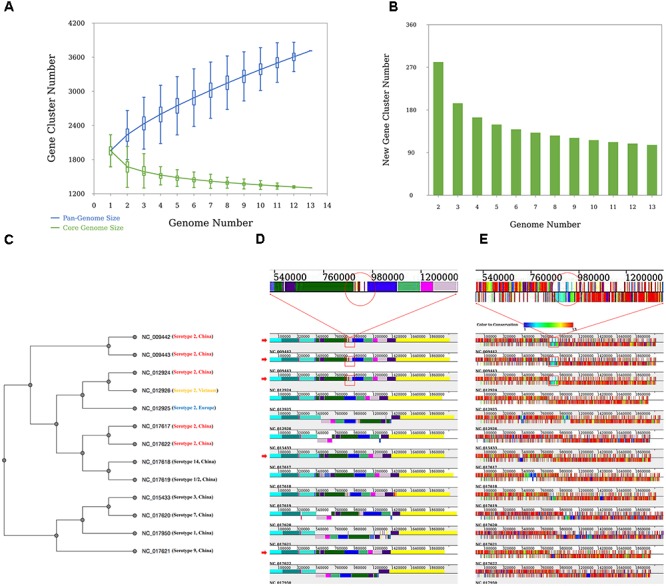
Results of the case study. **(A)** Pan-genome profiles of 13 *Streptococcus suis* strains. Pan- and core-genome size prediction with all combinations of studied strains. **(B)** New prediction happens when an additional genome is introduced. **(C)** Phylogenetic tree based on UPGMA built from 13 *S. suis* genomes. All strains are labeled with serotype and place of origin. All Chinese serotype two strains are marked in red, Vietnamese serotype two strains in yellow, and European serotype two strains in blue. **(D)** Alignment result of 13 *S. suis* genomes. The red arrow indicates Chinese serotype 2 *S. suis* strains. Red rectangles indicate the ∼89-kb PAI (candidate pathogenicity island) proposed by Chen et al. (2007). **(E)** Orthologous gene distribution of 13 *S. suis* genomes. Different colors represent degrees of conservation for each gene (PGAP-X).

The analysis revealed that *S. suis* has an open pan-genome, whose pan-genome increases in number by 110 genes when a newly sequenced genome is added (**Figures 3A,B**). The phylogenetic results give us a clear idea of the phylogenetic relationship among different serotypes of the 13 *S. suis* strains. Taking one of the phylogenetic trees (UPGMA tree based on the presence or absence of orthologous genes) as an example (**Figure 3C**), the 13 strains are clearly grouped into two subgroups and all the serotype 2 strains are tightly grouped in the same clade. Serotype 1, 3, 7, and 9 strains could be assigned to a common clade. Serotype 14 and 1/2 strains were more closely related to the serotype 2 strains than to the other four serotypes. This result is consistent with earlier findings by Zhang et al. (2011). From genome structure conservation analysis (**Figure 3D**), the 89K PAI exists in three serotype 2 strains isolated in China (NC_009442, NC_009443, and NC_012924), while the other two Chinese serotype 2 strains (NC_017617 and NC_017622) harbored on different branches of the phylogenetic tree (**Figure 3C**) do not contain this PAI. This result confirms an earlier observation that the 89K PAI is prevalent and specific for Chinese serotype 2 virulent strains (Chen et al., 2007). The genes on the PAI are dispensable genes with lower-grade conservation (**Figure 3E**). This suggests that these genes located in a cluster might have been introduced through HGT or homologous recombination with other closely related species (Zhang et al., 2011).

## Discussion

Since the term of microbial pan-genome was introduced in 2005, several stand-alone and web-based tools have been developed for pan-genome analyses (**Supplementary Table S1**). However, stand-alone tools always require basic programming skills for installation and operation. We developed PGAweb for in-depth inference and visualization in bacterial pan-genome analysis. PGAweb has a user-friendly, web-based interface, one-click input data submission, and smooth and efficient data analysis. No installation, command lines, or informatics skills are necessary for using PGAweb, making it very efficient for users, especially experimental scientists. PGAweb provides various interactive and customizable functions including orthologous clustering, pan-genome profiling, sequence variation, evolutionary analysis, and functional classification. Different visualization methods are used for enhanced genomic structural dynamics and sequence diversity display. These are helpful for intuitively understanding the dynamics and evolution of bacterial genomes. According to the pan-genome concept, three genomic data-sets is the minimum number for pan-genome analysis. In the case of technical and resource limitations, PGAweb is only suitable for performing analyzes on the pan-genome of a moderate number of strains (no more than 50 strains). The key issue for large scale pan-genome analysis is the input data uploading with limited network speed. We estimate that the input data size will be exceed 500 Mb for 50 strains pan-genome analysis, which will cost users a very long time for input data uploading. Furthermore, many factors may influence the runtime of PGAweb, such as the number input data sets, genome sizes, selected method, etc. The running time grows exponentially with the increase in number of input data sets due to the computational complexity (**Supplementary Figure S1**). The current version of PGAweb still has some shortcomings, which only supports the pan-genome analysis of bacterial and archaeal genomes so far. With the development of sequencing technology, the pan-genome concept has also been widely utilized to perform comparative genomic analysis in fungi (Dunn et al., 2012) and plants (Cao et al., 2011; Li et al., 2014; Golicz et al., 2016). In a future version, we plan to develop a new algorithm to support the analysis of eukaryotes, especially plants and animals, and integrate more efficient methods for large scale pan-genome analysis.

## Conclusion

Here, we present a fast and freely available online server, PGAweb, with integration of our previously published software, PGAP, PanGP, and PGAP-X. In brief, PGAweb has a user-friendly web interface, one-click input data submission, and smooth and efficient data analysis, and provides not only diverse methods for identification of orthologous genes but also powerful tools to display genome dynamics and structure characteristics. Case study indicates that PGAweb can intuitively reflect the characteristics of bacterial pan-genomes such as an open or closed pan-genome. Orthologous clustering, gene distribution based on conservation, genome structure conservation, and genetic variation, as well as their visualization, are all helpful for biologists to study bacterial genome dynamics to identify pathogenic and drug-resistant genes and their sequence contexts.

## Author Contributions

JX, ZJ, and JAW conceived and designed the web server. XC performed the server front-end and back-end. YAZ carried out the case studies. YOZ, CS, MY, JNW, QL, BZ, and MC contributed to the web server testing. YAZ, ZZ, and JX wrote the paper. JY polished the language and gave many constructive suggestions.

## Conflict of Interest Statement

The authors declare that the research was conducted in the absence of any commercial or financial relationships that could be construed as a potential conflict of interest.

## Footnotes

1 http://www.bootcss.com/

2 https://d3js.org/

3 http://echarts.baidu.com/

4 http://expressjs.com/

5 https://www.mongodb.com/

6 https://www.docker.com/

7 ftp://ftp.ncbi.nlm.nih.gov/genomes/archive/old_genbank/Bacteria/

## Supplementary Material

FIGURE S1 The curve of input data sets and running time. Here the running time refers to PGAweb analysis time and do not contain the input data uploading time.

TABLE The function comparation of online pan-genome analysis tools.

## References

[B1] Alexeyenko A. Tamas I. Liu G. Sonnhammer E. L. (2006). Automatic clustering of orthologs and inparalogs shared by multiple proteomes. *Bioinformatics* 22:e9-15. 10.1093/bioinformatics/btl213 16873526

[B2] Bayjanov J. R. Siezen R. J. van Hijum S. A. (2010). PanCGHweb: a web tool for genotype calling in pangenome CGH data. *Bioinformatics* 26 1256–1257. 10.1093/bioinformatics/btq103 20219865 PMC2859125

[B3] Benedict M. N. Henriksen J. R. Metcalf W. W. Whitaker R. J. Price N. D. (2014). ITEP: an integrated toolkit for exploration of microbial pan-genomes. *BMC Genomics* 15:8. 10.1186/1471-2164-15-8 24387194 PMC3890548

[B4] Brittnacher M. J. Fong C. Hayden H. S. Jacobs M. A. Radey M. Rohmer L. (2011). PGAT: a multistrain analysis resource for microbial genomes. *Bioinformatics* 27 2429–2430. 10.1093/bioinformatics/btr418 21765097 PMC3157930

[B5] Cao J. Schneeberger K. Ossowski S. Gunther T. Bender S. Fitz J. (2011). Whole-genome sequencing of multiple *Arabidopsis thaliana* populations. *Nat. Genet.* 43 956–963. 10.1038/ng.911 21874002

[B6] Chen C. Tang J. Dong W. Wang C. Feng Y. Wang J. (2007). A glimpse of streptococcal toxic shock syndrome from comparative genomics of *S. suis* 2 Chinese isolates. *PLoS One* 2:e315. 10.1371/journal.pone.0000315 17375201 PMC1820848

[B7] Contreras-Moreira B. Vinuesa P. (2013). GET_HOMOLOGUES, a versatile software package for scalable and robust microbial pangenome analysis. *Appl. Environ. Microbiol.* 79 7696–7701. 10.1128/aem.02411-13 24096415 PMC3837814

[B8] Darling A. E. Mau B. Perna N. T. (2010). ProgressiveMauve: multiple genome alignment with gene gain, loss and rearrangement. *PLoS One* 5:e11147. 10.1371/journal.pone.0011147 20593022 PMC2892488

[B9] D’Auria G. Jimenez-Hernandez N. Peris-Bondia F. Moya A. Latorre A. (2010). *Legionella pneumophila* pangenome reveals strain-specific virulence factors. *BMC Genomics* 11:181. 10.1186/1471-2164-11-181 20236513 PMC2859405

[B10] Ding W. Baumdicker F. Neher R. A. (2018). panX: pan-genome analysis and exploration. *Nucleic Acids Res.* 46:e5. 10.1093/nar/gkx977 29077859 PMC5758898

[B11] Donati C. Hiller N. L. Tettelin H. Muzzi A. Croucher N. J. Angiuoli S. V. (2010). Structure and dynamics of the pan-genome of *Streptococcus pneumoniae* and closely related species. *Genome Biol.* 11:R107. 10.1186/gb-2010-11-10-r107 21034474 PMC3218663

[B12] Dunn B. Richter C. Kvitek D. J. Pugh T. Sherlock G. (2012). Analysis of the *Saccharomyces cerevisiae* pan-genome reveals a pool of copy number variants distributed in diverse yeast strains from differing industrial environments. *Genome Res.* 22 908–924. 10.1101/gr.130310.111 22369888 PMC3337436

[B13] Enright A. J. Van Dongen S. Ouzounis C. A. (2002). An efficient algorithm for large-scale detection of protein families. *Nucleic Acids Res.* 30 1575–1584. 10.1093/nar/30.7.1575 11917018 PMC101833

[B14] Felsenstein J. (1989). Mathematical evolutionary-theory - Feldman,Mw. *Science* 246 941–942. 10.1126/science.246.4932.941 17812579

[B15] Golicz A. A. Bayer P. E. Barker G. C. Edger P. P. Kim H. Martinez P. A. (2016). The pangenome of an agronomically important crop plant *Brassica oleracea*. *Nat. Commun.* 7:13390. 10.1038/ncomms13390 27834372 PMC5114598

[B16] Hu P. Yang M. Zhang A. Wu J. Chen B. Hua Y. (2011). Comparative genomics study of multi-drug-resistance mechanisms in the antibiotic-resistant *Streptococcus suis* R61 strain. *PLoS One* 6:e24988. 10.1371/journal.pone.0024988 21966396 PMC3180280

[B17] Katoh K. Toh H. (2010). Parallelization of the MAFFT multiple sequence alignment program. *Bioinformatics* 26 1899–1900. 10.1093/bioinformatics/btq224 20427515 PMC2905546

[B18] Lefebure T. Stanhope M. J. (2007). Evolution of the core and pan-genome of *Streptococcus*: positive selection, recombination, and genome composition. *Genome Biol.* 8:R71. 10.1186/gb-2007-8-5-r71 17475002 PMC1929146

[B19] Li Y. H. Zhou G. Y. Ma J. X. Jiang W. K. Jin L. G. Zhang Z. H. (2014). De novo assembly of soybean wild relatives for pan-genome analysis of diversity and agronomic traits. *Nat. Biotechnol.* 32 1045–1052. 10.1038/nbt.2979 25218520

[B20] Maione D. Margarit I. Rinaudo C. D. Masignani V. Mora M. Scarselli M. (2005). Identification of a universal Group B *Streptococcus* vaccine by multiple genome screen. *Science* 309 148–150. 10.1126/science.1109869 15994562 PMC1351092

[B21] Ostlund G. Schmitt T. Forslund K. Kostler T. Messina D. N. Roopra S. (2010). InParanoid 7: new algorithms and tools for eukaryotic orthology analysis. *Nucleic Acids Res.* 38 D196–D203. 10.1093/nar/gkp931 19892828 PMC2808972

[B22] Page A. J. Cummins C. A. Hunt M. Wong V. K. Reuter S. Holden M. T. (2015). Roary: rapid large-scale prokaryote pan genome analysis. *Bioinformatics* 31 3691–3693. 10.1093/bioinformatics/btv421 26198102 PMC4817141

[B23] Pantoja Y. Pinheiro K. Veras A. Araujo F. Lopes de Sousa A., (2017). PanWeb: a web interface for pan-genomic analysis. *PLoS One* 12:e0178154. 10.1371/journal.pone.0178154 28542514 PMC5443543

[B24] Rasko D. A. Rosovitz M. J. Myers G. S. Mongodin E. F. Fricke W. F. Gajer P. (2008). The pangenome structure of *Escherichia coli*: comparative genomic analysis of *E. coli* commensal and pathogenic isolates. *J. Bacteriol.* 190 6881–6893. 10.1128/jb.00619-08 18676672 PMC2566221

[B25] Remm M. Storm C. E. Sonnhammer E. L. (2001). Automatic clustering of orthologs and in-paralogs from pairwise species comparisons. *J. Mol. Biol.* 314 1041–1052. 10.1006/jmbi.2000.5197 11743721

[B26] Saenz H. L. Engel P. Stoeckli M. C. Lanz C. Raddatz G. Vayssier-Taussat M. (2007). Genomic analysis of *Bartonella* identifies type IV secretion systems as host adaptability factors. *Nat. Genet.* 39 1469–1476. 10.1038/ng.2007.38 18037886

[B27] Santos A. R. Barbosa E. Fiaux K. Zurita-Turk M. Chaitankar V. Kamapantula B. (2013). PANNOTATOR: an automated tool for annotation of pan-genomes. *Genet. Mol. Res.* 12 2982–2989. 10.4238/2013.August.16.2 24065654

[B28] Serruto D. Serino L. Masignani V. Pizza M. (2009). Genome-based approaches to develop vaccines against bacterial pathogens. *Vaccine* 27 3245–3250. 10.1016/j.vaccine.2009.01.072 19200820

[B29] Staats J. J. Feder I. Okwumabua O. Chengappa M. M. (1997). *Streptococcus suis*: past and present. *Vet. Res. Commun.* 21 381–407. 10.1023/A:1005870317757 9266659

[B30] Sugawara M. Epstein B. Badgley B. D. Unno T. Xu L. Reese J. (2013). Comparative genomics of the core and accessory genomes of 48 *Sinorhizobium* strains comprising five genospecies. *Genome Biol.* 14:R17. 10.1186/gb-2013-14-2-r17 23425606 PMC4053727

[B31] Tatusov R. L. Galperin M. Y. Natale D. A. Koonin E. V. (2000). The COG database: a tool for genome-scale analysis of protein functions and evolution. *Nucleic Acids Res.* 28 33–36. 10.1093/nar/28.1.33 10592175 PMC102395

[B32] Tettelin H. Masignani V. Cieslewicz M. J. Donati C. Medini D. Ward N. L. (2005). Genome analysis of multiple pathogenic isolates of *Streptococcus agalactiae*: implications for the microbial “pan-genome”. *Proc. Natl. Acad. Sci. U.S.A.* 102 13950–13955. 10.1073/pnas.0506758102 16172379 PMC1216834

[B33] Tian X. Zhang Z. Yang T. Chen M. Li J. Chen F. (2016). Comparative genomics analysis of *Streptomyces* species reveals their adaptation to the marine environment and their diversity at the genomic level. *Front. Microbiol.* 7:998. 10.3389/fmicb.2016.00998 27446038 PMC4921485

[B34] Van Dongen S. (2008). Graph clustering via a discrete uncoupling process. *SIAM J. Matrix Anal. Appl.* 30 121–141. 10.1137/040608635

[B35] Vernikos G. Medini D. Riley D. R. Tettelin H. (2015). Ten years of pan-genome analyses. *Curr. Opin. Microbiol.* 23 148–154. 10.1016/j.mib.2014.11.016 25483351

[B36] Xiao J. Zhang Z. Wu J. Yu J. (2015). A brief review of software tools for pangenomics. *Genomics Proteomics Bioinformatics* 13 73–76. 10.1016/j.gpb.2015.01.007 25721608 PMC4411478

[B37] Zekic T. Holley G. Stoye J. (2018). Pan-genome storage and analysis techniques. *Methods Mol. Biol.* 1704 29–53. 10.1007/978-1-4939-7463-4_2 29277862

[B38] Zhang A. Yang M. Hu P. Wu J. Chen B. Hua Y. (2011). Comparative genomic analysis of *Streptococcus suis* reveals significant genomic diversity among different serotypes. *BMC Genomics* 12:523. 10.1186/1471-2164-12-523 22026465 PMC3227697

[B39] Zhao Y. Jia X. Yang J. Ling Y. Zhang Z. Yu J. (2014). PanGP: a tool for quickly analyzing bacterial pan-genome profile. *Bioinformatics* 30 1297–1299. 10.1093/bioinformatics/btu017 24420766 PMC3998138

[B40] Zhao Y. Sun C. Zhao D. Zhang Y. You Y. Jia X. (2018). PGAP-X: extension on pan-genome analysis pipeline. *BMC Genomics* 19(Suppl. 1):36. 10.1186/s12864-017-4337-7 29363431 PMC5780747

[B41] Zhao Y. Wu J. Yang J. Sun S. Xiao J. Yu J. (2012). PGAP: pan-genomes analysis pipeline. *Bioinformatics* 28 416–418. 10.1093/bioinformatics/btr655 22130594 PMC3268234

