# Supplementary figures and images for: PGAweb: A Web Server for Bacterial Pan-Genome Analysis
Source: Front Microbiol. 2018 Aug 21;9:1910. doi: 10.3389/fmicb.2018.01910 (PMC6110895; doi:10.3389/fmicb.2018.01910)

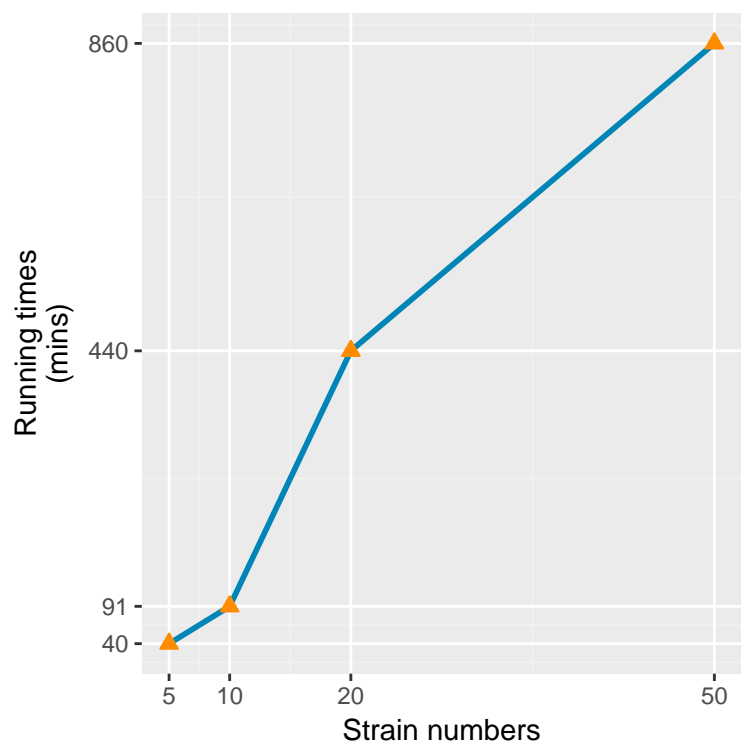

Supplement: FIGURE S1 — The curve of input data sets and running time. Here the running time refers to PGAweb analysis time and do not contain the input data uploading time. [file Data_Sheet_1.PDF]
