# Supplementary material for: PGAweb: A Web Server for Bacterial Pan-Genome Analysis
Source: Front Microbiol. 2018 Aug 21;9:1910. doi: 10.3389/fmicb.2018.01910 (PMC6110895; doi:10.3389/fmicb.2018.01910)
Supplement: TABLE — The function comparation of online pan-genome analysis tools. [file Table_1.DOCX]

|  | Spine and AGEnt | PANNOTATOR | PanCGH  web | PGAT | Panseq | PanWeb | PGAweb |
| --- | --- | --- | --- | --- | --- | --- | --- |
| Clustering homologous genes | 🗸 | 🗸 | 🗸 | 🗸 | 🗸 | 🗸 | 🗸 |
| Identifying SNPs |  |  |  | 🗸 | 🗸 | 🗸 | 🗸 |
| Plotting pangenomic profiles |  |  |  |  |  | 🗸 | 🗸 |
| Genotyping |  |  | 🗸 |  |  |  |  |
| Phylogenetic analysis |  |  | 🗸 |  |  | 🗸 | 🗸 |
| Function-based searching or analysis |  |  |  | 🗸 |  | 🗸 | 🗸 |
| Annotation and/or curation |  | 🗸 |  |  |  | ­­­ |  |
| Whole genome alignment |  |  |  |  |  |  | 🗸 |
| Genetic variation visualization |  |  |  |  |  |  | 🗸 |
| Gene conservation level visualization |  |  |  |  |  |  | 🗸 |
| Pathway analysis |  |  |  | 🗸 |  |  |  |

**Supplementary table 1** The function comparation of online pan-genome analysis tools
